# Retraction of “Nanogrooved Elastomeric Diaphragm Arrays for Assessment of Cardiomyocytes under Synergistic Effects of Circular Mechanical Stimuli and Electrical Conductivity to Enhance Intercellular Communication”

**DOI:** 10.1021/acsbiomaterials.5c00906

**Published:** 2025-05-27

**Authors:** Abdullah-Bin Siddique, Keith A. Williams, Nathan S. Swami

The authors retract this article due to an error in immunofluorescence data analysis arising from not accounting for metal-enhanced fluorescence effects from embedded silver nanowires within the nanogrooved elastomeric diaphragms, which distorted the reported fluorescence intensity levels that were used to quantify protein expression levels of α-actinin and connexin-43. As a result, the reported inference of significantly increased fluorescence signal intensities that are attributed to enhanced connexin-43 and α-actinin expression levels for the cardiomyocytes cultured on silver nanowire-embedded nanogrooved substrates under mechanical stimulation is no longer justified.

Since the protein expression data was central to the conclusions, the authors retract this article to ensure the error does not mislead the scientific community in this field.

The original article was published on December 16, 2024, and retracted on May 27, 2025.

